# 3-[4-(3,4-Dimethyl-5,5-dioxo-2*H*,4*H*-pyrazolo­[4,3-*c*][1,2]benzothia­zin-2-yl)phen­yl]-2-hy­droxy-1-mesitylprop-2-en-1-one hexane hemisolvate

**DOI:** 10.1107/S1600536812000712

**Published:** 2012-01-18

**Authors:** Mujahid Hussain Bukhari, Matloob Ahmad, Hamid Latif Siddiqui, Salman Gul, Masood Parvez

**Affiliations:** a Institute of Chemistry, University of the Punjab, Lahore 54590, Pakistan; b Department of Chemistry, The University of Calgary, 2500 University Drive NW, Calgary, Alberta, Canada T2N 1N4

## Abstract

In the title compound, C_29_H_27_N_3_O_4_S·0.5C_6_H_14_, the heterocyclic thia­zine ring adopts a half-chair conformation with the S and N atoms displaced by 0.500 (5) and 0.229 (5) Å, respectively, on opposite sides from the mean plane formed by the remaining ring atoms. The mean planes of the pyrazole ring and the benzene ring bonded to it form a dihedral angle of 35.76 (11)° and an intra­molecular O—H⋯O hydrogen bond ocurs. The crystal structure features O—H⋯O and C—H⋯O hydrogen bonds. There is a half-mol­ecule of hexane in the asymmetric unit lying about an inversion center. It is disordered over two sets of sites with occupancy factors 0.590 (9) and 0.410 (9).

## Related literature

For the synthesis and biological activity of benzothia­zine derivatives, see: Ahmad *et al.* (2010 ▶); Siddiqui *et al.* (2007 ▶). For related structures, see: Siddiqui *et al.* (2008 ▶); Bukhari *et al.* (2008 ▶). For the preparation of the chalcone, see: Furniss *et al.* (1989 ▶).
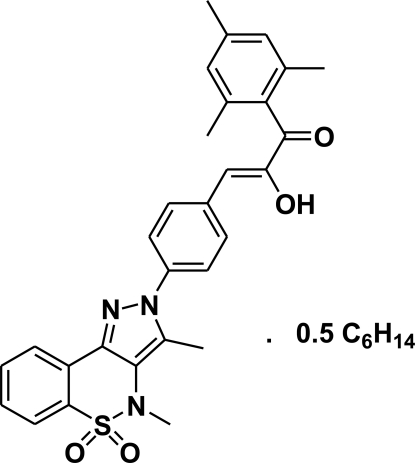


## Experimental

### 

#### Crystal data

C_29_H_27_N_3_O_4_S·0.5C_6_H_14_
*M* *_r_* = 556.68
Monoclinic, 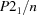

*a* = 7.1772 (2) Å
*b* = 23.2178 (5) Å
*c* = 16.7740 (4) Å
β = 99.526 (1)°
*V* = 2756.65 (12) Å^3^
*Z* = 4
Cu *K*α radiation
μ = 1.39 mm^−1^
*T* = 173 K
0.20 × 0.05 × 0.04 mm

#### Data collection

Bruker APEXII CCD diffractometer
Absorption correction: multi-scan (*SADABS*; Bruker, 2004 ▶) *T*_min_ = 0.768, *T*_max_ = 0.946
28059 measured reflections
4979 independent reflections
4190 reflections with *I* > 2σ(*I*)
*R*_int_ = 0.029

#### Refinement

*R*[*F* ^2^ > 2σ(*F* ^2^)] = 0.057
*wR*(*F* ^2^) = 0.158
*S* = 1.04
4979 reflections
377 parameters
10 restraints
H-atom parameters constrained
Δρ_max_ = 1.21 e Å^−3^
Δρ_min_ = −0.66 e Å^−3^

### 

Data collection: *APEX2* (Bruker, 2004 ▶); cell refinement: *SAINT* (Bruker, 2004 ▶); data reduction: *SAINT*; program(s) used to solve structure: *SHELXS97* (Sheldrick, 2008 ▶); program(s) used to refine structure: *SHELXL97* (Sheldrick, 2008 ▶); molecular graphics: *ORTEP-3* (Farrugia, 1997 ▶); software used to prepare material for publication: *SHELXL97*.

## Supplementary Material

Crystal structure: contains datablock(s) global, I. DOI: 10.1107/S1600536812000712/fj2493sup1.cif

Structure factors: contains datablock(s) I. DOI: 10.1107/S1600536812000712/fj2493Isup2.hkl

Supplementary material file. DOI: 10.1107/S1600536812000712/fj2493Isup3.cml

Additional supplementary materials:  crystallographic information; 3D view; checkCIF report

## Figures and Tables

**Table 1:** Hydrogen-bond geometry (Å, °)

*D*—H⋯*A*	*D*—H	H⋯*A*	*D*⋯*A*	*D*—H⋯*A*
O3—H3*O*⋯O2^i^	0.84	2.15	2.854 (3)	141
O3—H3*O*⋯O4	0.84	2.18	2.646 (3)	115
C3—H3⋯O1^ii^	0.95	2.56	3.328 (3)	138
C23—H23⋯O1^iii^	0.95	2.58	3.436 (4)	150
C16—H16⋯O3	0.95	2.28	2.907 (3)	123

Symmetry codes: (i) 
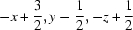
; (ii) 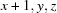; (iii) 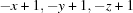.

**Fig. 1.**
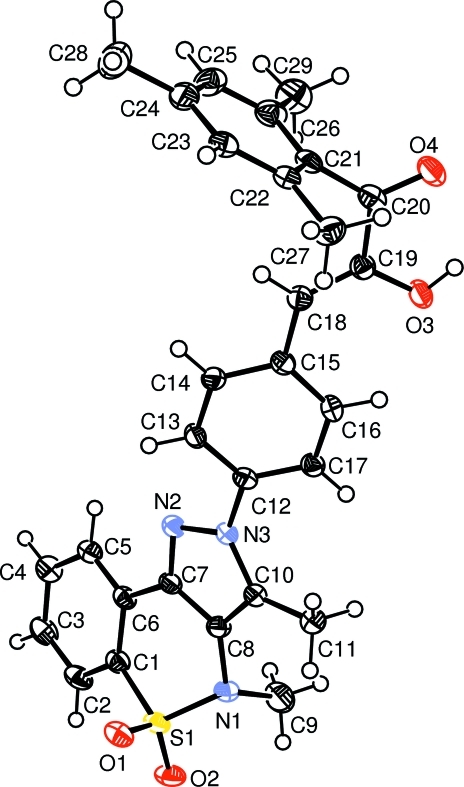
The title molecule with displacement ellipsoids plotted at 30% probability level (Farrugia, 1997).

**Fig. 2.**
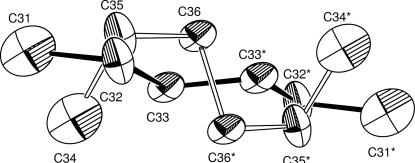
A plot of the disordered solvent molecule showing solid bonds between carbon atoms of the predominant fraction; H-atoms have been excluded for clarity. Symmetry operation: * = -x + 2, -y, -z + 1

## supplementary crystallographic information

### Comment

1,3-Diaryl prop-2-ene-1-ones, usually known as chalcones are generally used as starting materials for the synthesis of a variety of biologically active compounds (Siddiqui *et al.*, 2007). In continuation of our research project on potentially biologically active derivatives of benzothiazines (Ahmad *et al.*, 2010) and pyrimidines (Bukhari *et al.*, 2008), we report herein the crystal structure of the title compound.

The bond distances and angles in the title compound (Fig. 1) agree very well with the corresponding bond distances and angles reported in closely related compounds (Siddiqui *et al.*, 2008; Bukhari *et al.*, 2008). The heterocyclic thiazine ring adopts a half chair conformation with atoms S1 and N1 displaced by 0.500 (5) and 0.229 (5) Å, respectively, on the opposite sides from the mean plane formed by the remaining ring atoms. The mean planes of the pyrazolyl (N2/N3/C7/C8/C10) and benzene (C12–C17) rings form a dihedral angle 35.76 (11)°.

The structure is stabilized by O3—H3O···O2 intermolecular hydrogen bonds and further consolidated by C—H···O type hydrogen bonding interactions; intramolecular interactions of the type O—H···O and C—H···O are also present (Tab. 1).

### Experimental

The chalcone was synthesized by following a reported method (Furniss *et al.*, 1989). A mixture of 1-(4-(3,4-dimethyl-5,5-dioxidobenzo[*e*]pyrazolo[4,3-*c*][1,2]thiazin -2(4*H*)-yl)phenyl)benzaldehyde (10.0 mmol, 3.53 g), 1-mesitylethanone (10.0 mmol, 1.62 g), MeONa (10.0 mmol) in MeOH (10 ml) was stirred at ambient temperature for a period of two hours. The resulted yellow precipitates were collected and washed with MeOH followed by cold water. The product was purified by flash chromatography by eluting with CHCl_3_/MeOH (4:1). The resulted chalcone (5 mmol, 2.49 g) was dissolved in EtOH (10 ml) and refluxed for 30 minutes along with portion wise addition of 30% H_2_O_2_ (1.5 ml). The yellowish white precipitates formed were collected and washed with EtOH and then with pure water. Recrystallization from n-hexane/CHCl_3_ afforded pure yellow crystals of the title compound. Yield; 68%; m.p. 472–474 K.

### Refinement

There is a half molecule of hexane in the asymmetric unit lying about an inversion center that was disordered over two sites with occupancy factors 0.589 (9) and 0.411 (9) (Fig. 2). The C—C distances in the solvate were constrained at 1.54 (1) Å and EADP commands in *SHELXL* (Sheldrick, 2008) were used to apply constraints on the *U*_ij_ of the solvent C-atoms. The H atoms were included at geometrically idealized positions and refined in riding-model approximation with the following constraints: O—H = 0.84, C—H = 0.95, 0.98 and 0.99 Å, for aryl, methyl and methylene H-atoms, respectively. The *U*_iso_(H) were allowed at 1.2*U*_eq_(C) or 1.5*U*_eq_(*O*/methyl C). The final difference map showed some residual electron density in the close proximity of the solvent molecule and was essentially meaningless.

### Crystal data

**Table:**

C_29_H_27_N_3_O_4_S·0.5C_6_H_14_	*F*(000) = 1180
*M**_r_* = 556.68	*D*_x_ = 1.341 Mg m^−^^3^
Monoclinic, *P*2_1_/*n*	Cu *K*α radiation, λ = 1.54178 Å
Hall symbol: -P 2yn	Cell parameters from 6317 reflections
*a* = 7.1772 (2) Å	θ = 3.3–68.1°
*b* = 23.2178 (5) Å	µ = 1.39 mm^−^^1^
*c* = 16.7740 (4) Å	*T* = 173 K
β = 99.526 (1)°	Needle, yellow
*V* = 2756.65 (12) Å^3^	0.20 × 0.05 × 0.04 mm
*Z* = 4	

### Data collection

**Table:**

Bruker APEXII CCD diffractometer	4979 independent reflections
Radiation source: fine-focus sealed tube	4190 reflections with *I* > 2σ(*I*)
graphite	*R*_int_ = 0.029
ω and φ scans	θ_max_ = 68.1°, θ_min_ = 3.3°
Absorption correction: multi-scan (*SADABS*; Bruker, 2004)	*h* = −8→8
*T*_min_ = 0.768, *T*_max_ = 0.946	*k* = −27→26
28059 measured reflections	*l* = −17→20

### Refinement

**Table:**

Refinement on *F*^2^	Primary atom site location: structure-invariant direct methods
Least-squares matrix: full	Secondary atom site location: difference Fourier map
*R*[*F*^2^ > 2σ(*F*^2^)] = 0.057	Hydrogen site location: inferred from neighbouring sites
*wR*(*F*^2^) = 0.158	H-atom parameters constrained
*S* = 1.04	*w* = 1/[σ^2^(*F*_o_^2^) + (0.0769*P*)^2^ + 4.2798*P*] where *P* = (*F*_o_^2^ + 2*F*_c_^2^)/3
4979 reflections	(Δ/σ)_max_ = 0.003
377 parameters	Δρ_max_ = 1.21 e Å^−^^3^
10 restraints	Δρ_min_ = −0.66 e Å^−^^3^

### Special details

**Table:**

Geometry. All e.s.d.'s (except the e.s.d. in the dihedral angle between two l.s. planes) are estimated using the full covariance matrix. The cell e.s.d.'s are taken into account individually in the estimation of e.s.d.'s in distances, angles and torsion angles; correlations between e.s.d.'s in cell parameters are only used when they are defined by crystal symmetry. An approximate (isotropic) treatment of cell e.s.d.'s is used for estimating e.s.d.'s involving l.s. planes.
Refinement. Refinement of *F*^2^ against ALL reflections. The weighted *R*-factor *wR* and goodness of fit *S* are based on *F*^2^, conventional *R*-factors *R* are based on *F*, with *F* set to zero for negative *F*^2^. The threshold expression of *F*^2^ > σ(*F*^2^) is used only for calculating *R*-factors(gt) *etc*. and is not relevant to the choice of reflections for refinement. *R*-factors based on *F*^2^ are statistically about twice as large as those based on *F*, and *R*- factors based on ALL data will be even larger.

### Fractional atomic coordinates and isotropic or equivalent isotropic displacement parameters (Å^2^)

**Table:**

	*x*	*y*	*z*	*U*_iso_*/*U*_eq_	Occ. (<1)
S1	1.13474 (10)	0.58533 (3)	0.20982 (4)	0.02532 (19)	
O1	1.0238 (3)	0.62094 (8)	0.25325 (12)	0.0302 (5)	
O2	1.1891 (3)	0.60647 (8)	0.13687 (12)	0.0332 (5)	
O3	0.4765 (3)	0.20587 (8)	0.44658 (11)	0.0285 (4)	
H3O	0.4255	0.1733	0.4458	0.043*	
O4	0.3515 (3)	0.14501 (8)	0.56022 (13)	0.0350 (5)	
N1	1.0174 (3)	0.52495 (10)	0.18792 (13)	0.0285 (5)	
N2	1.0892 (3)	0.45865 (9)	0.38601 (13)	0.0222 (5)	
N3	0.9178 (3)	0.43723 (9)	0.35111 (13)	0.0217 (5)	
C1	1.3383 (4)	0.56538 (11)	0.27865 (16)	0.0252 (6)	
C2	1.5067 (4)	0.59522 (12)	0.27961 (18)	0.0304 (6)	
H2	1.5181	0.6231	0.2392	0.037*	
C3	1.6571 (4)	0.58368 (12)	0.34021 (19)	0.0327 (7)	
H3	1.7718	0.6045	0.3424	0.039*	
C4	1.6416 (4)	0.54185 (12)	0.39796 (18)	0.0295 (6)	
H4	1.7452	0.5346	0.4399	0.035*	
C5	1.4761 (4)	0.51059 (11)	0.39475 (17)	0.0251 (6)	
H5	1.4689	0.4809	0.4330	0.030*	
C6	1.3202 (4)	0.52247 (11)	0.33576 (16)	0.0225 (5)	
C7	1.1412 (4)	0.49174 (11)	0.32872 (16)	0.0221 (5)	
C8	1.0034 (4)	0.49272 (11)	0.25929 (16)	0.0240 (6)	
C9	1.0698 (7)	0.49052 (15)	0.1201 (2)	0.0551 (11)	
H9A	0.9775	0.4595	0.1059	0.083*	
H9B	1.1957	0.4738	0.1366	0.083*	
H9C	1.0706	0.5155	0.0730	0.083*	
C10	0.8590 (4)	0.45768 (11)	0.27444 (16)	0.0239 (6)	
C11	0.6749 (4)	0.44530 (13)	0.22205 (17)	0.0313 (6)	
H11A	0.6405	0.4776	0.1849	0.047*	
H11B	0.5771	0.4401	0.2558	0.047*	
H11C	0.6859	0.4101	0.1909	0.047*	
C12	0.8229 (3)	0.39903 (11)	0.39740 (16)	0.0210 (5)	
C13	0.8406 (4)	0.40655 (11)	0.48052 (16)	0.0230 (6)	
H13	0.9112	0.4380	0.5062	0.028*	
C14	0.7546 (4)	0.36800 (11)	0.52546 (16)	0.0224 (5)	
H14	0.7668	0.3733	0.5822	0.027*	
C15	0.6494 (3)	0.32111 (11)	0.48915 (16)	0.0211 (5)	
C16	0.6317 (4)	0.31543 (11)	0.40510 (16)	0.0232 (5)	
H16	0.5587	0.2847	0.3788	0.028*	
C17	0.7180 (4)	0.35349 (11)	0.35994 (16)	0.0227 (5)	
H17	0.7057	0.3486	0.3031	0.027*	
C18	0.5692 (4)	0.28073 (11)	0.54079 (16)	0.0226 (5)	
H18	0.5722	0.2927	0.5952	0.027*	
C19	0.4912 (4)	0.22864 (11)	0.52148 (16)	0.0238 (6)	
C20	0.4184 (4)	0.19208 (11)	0.58181 (17)	0.0252 (6)	
C21	0.4307 (4)	0.21365 (11)	0.66674 (16)	0.0251 (6)	
C22	0.2874 (4)	0.24899 (11)	0.68694 (16)	0.0252 (6)	
C23	0.3056 (4)	0.27042 (12)	0.76527 (17)	0.0282 (6)	
H23	0.2093	0.2946	0.7794	0.034*	
C24	0.4618 (4)	0.25727 (13)	0.82337 (17)	0.0331 (7)	
C25	0.5993 (4)	0.22111 (14)	0.80197 (18)	0.0356 (7)	
H25	0.7050	0.2113	0.8416	0.043*	
C26	0.5871 (4)	0.19889 (13)	0.72447 (18)	0.0309 (6)	
C27	0.1172 (4)	0.26465 (13)	0.62553 (17)	0.0314 (6)	
H27A	0.0241	0.2843	0.6526	0.047*	
H27B	0.0611	0.2296	0.5993	0.047*	
H27C	0.1556	0.2903	0.5848	0.047*	
C28	0.4801 (5)	0.28102 (17)	0.90764 (19)	0.0476 (9)	
H28A	0.5311	0.2512	0.9466	0.071*	
H28B	0.3556	0.2931	0.9181	0.071*	
H28C	0.5656	0.3142	0.9131	0.072*	
C29	0.7408 (4)	0.16001 (15)	0.7048 (2)	0.0438 (8)	
H29A	0.7206	0.1209	0.7235	0.066*	
H29B	0.8637	0.1742	0.7318	0.066*	
H29C	0.7382	0.1596	0.6462	0.066*	
C31	0.803 (3)	0.1081 (5)	0.5070 (14)	0.217 (9)	0.590 (10)
H31A	0.7757	0.1441	0.4766	0.326*	0.590 (10)
H31B	0.8510	0.1170	0.5637	0.326*	0.590 (10)
H31C	0.6864	0.0854	0.5034	0.326*	0.590 (10)
C32	0.948 (3)	0.0742 (4)	0.4719 (11)	0.133 (5)	0.590 (10)
H32A	1.0731	0.0932	0.4809	0.159*	0.590 (10)
H32B	0.9081	0.0664	0.4135	0.159*	0.590 (10)
C33	0.9451 (14)	0.0198 (3)	0.5233 (6)	0.122 (5)	0.590 (10)
H33A	1.0099	0.0257	0.5795	0.146*	0.590 (10)
H33B	0.8150	0.0057	0.5235	0.146*	0.590 (10)
C34	0.955 (5)	0.0886 (9)	0.5440 (16)	0.217 (9)	0.410 (10)
H34A	0.9759	0.1303	0.5444	0.326*	0.410 (10)
H34B	1.0703	0.0691	0.5695	0.326*	0.410 (10)
H34C	0.8520	0.0798	0.5740	0.326*	0.410 (10)
C35	0.902 (4)	0.0682 (7)	0.4580 (14)	0.133 (5)	0.410 (10)
H35A	0.7733	0.0811	0.4337	0.159*	0.410 (10)
H35B	0.9935	0.0814	0.4239	0.159*	0.410 (10)
C36	0.9098 (19)	0.0039 (6)	0.4696 (12)	0.122 (5)	0.410 (10)
H36A	0.9175	−0.0162	0.4183	0.146*	0.410 (10)
H36B	0.7983	−0.0103	0.4914	0.146*	0.410 (10)

### Atomic displacement parameters (Å^2^)

**Table:**

	*U*^11^	*U*^22^	*U*^33^	*U*^12^	*U*^13^	*U*^23^
S1	0.0326 (4)	0.0185 (3)	0.0261 (4)	0.0007 (3)	0.0084 (3)	0.0052 (2)
O1	0.0337 (11)	0.0220 (10)	0.0367 (11)	0.0032 (8)	0.0109 (9)	0.0033 (8)
O2	0.0439 (12)	0.0270 (10)	0.0306 (11)	0.0030 (9)	0.0117 (9)	0.0109 (8)
O3	0.0361 (11)	0.0214 (10)	0.0290 (10)	−0.0087 (8)	0.0085 (8)	−0.0041 (8)
O4	0.0454 (12)	0.0229 (10)	0.0378 (12)	−0.0097 (9)	0.0099 (9)	−0.0008 (9)
N1	0.0407 (14)	0.0235 (12)	0.0218 (12)	−0.0029 (10)	0.0063 (10)	0.0045 (9)
N2	0.0236 (11)	0.0187 (11)	0.0242 (11)	−0.0022 (9)	0.0040 (9)	0.0012 (9)
N3	0.0249 (11)	0.0178 (10)	0.0221 (11)	−0.0026 (9)	0.0032 (9)	0.0010 (9)
C1	0.0306 (14)	0.0206 (13)	0.0259 (14)	0.0011 (11)	0.0094 (11)	0.0004 (11)
C2	0.0372 (16)	0.0221 (14)	0.0356 (16)	−0.0010 (12)	0.0165 (13)	0.0046 (12)
C3	0.0288 (15)	0.0274 (15)	0.0444 (18)	−0.0036 (12)	0.0131 (13)	0.0001 (13)
C4	0.0246 (14)	0.0283 (14)	0.0362 (16)	0.0001 (11)	0.0064 (12)	−0.0003 (12)
C5	0.0286 (14)	0.0206 (13)	0.0277 (14)	0.0001 (11)	0.0087 (11)	0.0019 (11)
C6	0.0269 (13)	0.0180 (12)	0.0242 (13)	−0.0004 (10)	0.0094 (11)	−0.0013 (10)
C7	0.0279 (14)	0.0162 (12)	0.0231 (13)	0.0007 (10)	0.0065 (11)	0.0007 (10)
C8	0.0309 (14)	0.0203 (13)	0.0210 (13)	0.0009 (11)	0.0052 (11)	0.0023 (10)
C9	0.108 (3)	0.0327 (18)	0.0291 (17)	−0.0122 (19)	0.0229 (19)	−0.0026 (14)
C10	0.0293 (14)	0.0186 (13)	0.0230 (13)	0.0007 (10)	0.0019 (11)	−0.0001 (10)
C11	0.0332 (15)	0.0283 (15)	0.0291 (15)	−0.0037 (12)	−0.0040 (12)	0.0033 (12)
C12	0.0208 (12)	0.0171 (12)	0.0254 (13)	0.0019 (10)	0.0045 (10)	0.0022 (10)
C13	0.0244 (13)	0.0172 (12)	0.0265 (14)	−0.0024 (10)	0.0018 (11)	−0.0016 (10)
C14	0.0270 (13)	0.0196 (12)	0.0205 (13)	0.0015 (10)	0.0035 (10)	0.0009 (10)
C15	0.0201 (12)	0.0192 (12)	0.0247 (13)	0.0013 (10)	0.0053 (10)	0.0002 (10)
C16	0.0234 (13)	0.0192 (12)	0.0270 (14)	−0.0026 (10)	0.0045 (11)	−0.0034 (11)
C17	0.0259 (13)	0.0196 (13)	0.0230 (13)	0.0013 (10)	0.0053 (10)	−0.0004 (10)
C18	0.0228 (13)	0.0229 (13)	0.0226 (13)	0.0005 (10)	0.0049 (10)	0.0004 (10)
C19	0.0231 (13)	0.0224 (13)	0.0259 (14)	0.0004 (10)	0.0042 (11)	−0.0001 (11)
C20	0.0220 (13)	0.0195 (13)	0.0335 (15)	0.0004 (10)	0.0035 (11)	0.0040 (11)
C21	0.0282 (14)	0.0192 (13)	0.0281 (14)	−0.0061 (11)	0.0052 (11)	0.0050 (11)
C22	0.0306 (14)	0.0200 (13)	0.0250 (14)	−0.0049 (11)	0.0049 (11)	0.0048 (11)
C23	0.0337 (15)	0.0252 (14)	0.0270 (14)	−0.0070 (12)	0.0087 (12)	0.0036 (11)
C24	0.0368 (16)	0.0372 (16)	0.0257 (15)	−0.0151 (13)	0.0067 (12)	0.0046 (12)
C25	0.0275 (15)	0.0454 (18)	0.0314 (16)	−0.0085 (13)	−0.0029 (12)	0.0106 (14)
C26	0.0239 (14)	0.0307 (15)	0.0371 (16)	−0.0047 (11)	0.0019 (12)	0.0088 (12)
C27	0.0334 (15)	0.0327 (15)	0.0270 (15)	0.0053 (12)	0.0014 (12)	0.0030 (12)
C28	0.050 (2)	0.064 (2)	0.0285 (17)	−0.0206 (18)	0.0037 (14)	−0.0018 (16)
C29	0.0319 (16)	0.0445 (19)	0.053 (2)	0.0068 (14)	0.0022 (15)	0.0049 (16)
C31	0.20 (2)	0.205 (18)	0.25 (2)	−0.049 (16)	0.053 (18)	0.100 (15)
C32	0.131 (11)	0.098 (5)	0.191 (11)	−0.030 (6)	0.092 (9)	−0.036 (7)
C33	0.078 (6)	0.076 (7)	0.196 (14)	−0.039 (5)	−0.021 (8)	0.007 (7)
C34	0.20 (2)	0.205 (18)	0.25 (2)	−0.049 (16)	0.053 (18)	0.100 (15)
C35	0.131 (11)	0.098 (5)	0.191 (11)	−0.030 (6)	0.092 (9)	−0.036 (7)
C36	0.078 (6)	0.076 (7)	0.196 (14)	−0.039 (5)	−0.021 (8)	0.007 (7)

### Geometric parameters (Å, °)

**Table:**

S1—O1	1.428 (2)	C18—C19	1.349 (4)
S1—O2	1.431 (2)	C18—H18	0.9500
S1—N1	1.645 (2)	C19—C20	1.481 (4)
S1—C1	1.767 (3)	C20—C21	1.499 (4)
O3—C19	1.351 (3)	C21—C26	1.398 (4)
O3—H3O	0.8400	C21—C22	1.401 (4)
O4—C20	1.224 (3)	C22—C23	1.391 (4)
N1—C8	1.429 (3)	C22—C27	1.506 (4)
N1—C9	1.489 (4)	C23—C24	1.392 (4)
N2—C7	1.331 (3)	C23—H23	0.9500
N2—N3	1.366 (3)	C24—C25	1.387 (5)
N3—C10	1.370 (3)	C24—C28	1.503 (4)
N3—C12	1.424 (3)	C25—C26	1.388 (4)
C1—C2	1.391 (4)	C25—H25	0.9500
C1—C6	1.403 (4)	C26—C29	1.504 (4)
C2—C3	1.381 (4)	C27—H27A	0.9800
C2—H2	0.9500	C27—H27B	0.9800
C3—C4	1.389 (4)	C27—H27C	0.9800
C3—H3	0.9500	C28—H28A	0.9800
C4—C5	1.386 (4)	C28—H28B	0.9800
C4—H4	0.9500	C28—H28C	0.9800
C5—C6	1.393 (4)	C29—H29A	0.9800
C5—H5	0.9500	C29—H29B	0.9800
C6—C7	1.457 (4)	C29—H29C	0.9800
C7—C8	1.398 (4)	C31—C32	1.502 (10)
C8—C10	1.374 (4)	C31—H31A	0.9800
C9—H9A	0.9800	C31—H31B	0.9800
C9—H9B	0.9800	C31—H31C	0.9800
C9—H9C	0.9800	C32—C33	1.531 (9)
C10—C11	1.489 (4)	C32—H32A	0.9900
C11—H11A	0.9800	C32—H32B	0.9900
C11—H11B	0.9800	C33—C33^i^	1.509 (10)
C11—H11C	0.9800	C33—H33A	0.9900
C12—C17	1.387 (4)	C33—H33B	0.9900
C12—C13	1.390 (4)	C34—C35	1.507 (10)
C13—C14	1.380 (4)	C34—H34A	0.9800
C13—H13	0.9500	C34—H34B	0.9800
C14—C15	1.405 (4)	C34—H34C	0.9800
C14—H14	0.9500	C35—C36	1.504 (10)
C15—C16	1.401 (4)	C35—H35A	0.9900
C15—C18	1.458 (4)	C35—H35B	0.9900
C16—C17	1.376 (4)	C36—C36^i^	1.520 (10)
C16—H16	0.9500	C36—H36A	0.9900
C17—H17	0.9500	C36—H36B	0.9900
			
O1—S1—O2	119.43 (12)	O3—C19—C20	115.4 (2)
O1—S1—N1	107.41 (12)	O4—C20—C19	118.4 (3)
O2—S1—N1	107.64 (12)	O4—C20—C21	122.5 (2)
O1—S1—C1	106.79 (12)	C19—C20—C21	119.1 (2)
O2—S1—C1	109.62 (13)	C26—C21—C22	120.9 (3)
N1—S1—C1	105.05 (12)	C26—C21—C20	119.4 (3)
C19—O3—H3O	109.5	C22—C21—C20	119.7 (2)
C8—N1—C9	114.9 (2)	C23—C22—C21	118.7 (3)
C8—N1—S1	111.31 (18)	C23—C22—C27	119.9 (3)
C9—N1—S1	116.3 (2)	C21—C22—C27	121.4 (2)
C7—N2—N3	103.9 (2)	C22—C23—C24	121.4 (3)
N2—N3—C10	113.1 (2)	C22—C23—H23	119.3
N2—N3—C12	118.1 (2)	C24—C23—H23	119.3
C10—N3—C12	128.8 (2)	C25—C24—C23	118.5 (3)
C2—C1—C6	121.6 (3)	C25—C24—C28	120.7 (3)
C2—C1—S1	120.0 (2)	C23—C24—C28	120.9 (3)
C6—C1—S1	118.2 (2)	C24—C25—C26	122.0 (3)
C3—C2—C1	118.9 (3)	C24—C25—H25	119.0
C3—C2—H2	120.5	C26—C25—H25	119.0
C1—C2—H2	120.5	C25—C26—C21	118.5 (3)
C2—C3—C4	120.4 (3)	C25—C26—C29	119.6 (3)
C2—C3—H3	119.8	C21—C26—C29	121.9 (3)
C4—C3—H3	119.8	C22—C27—H27A	109.5
C5—C4—C3	120.4 (3)	C22—C27—H27B	109.5
C5—C4—H4	119.8	H27A—C27—H27B	109.5
C3—C4—H4	119.8	C22—C27—H27C	109.5
C4—C5—C6	120.4 (3)	H27A—C27—H27C	109.5
C4—C5—H5	119.8	H27B—C27—H27C	109.5
C6—C5—H5	119.8	C24—C28—H28A	109.5
C5—C6—C1	118.1 (2)	C24—C28—H28B	109.5
C5—C6—C7	123.7 (2)	H28A—C28—H28B	109.5
C1—C6—C7	118.2 (2)	C24—C28—H28C	109.5
N2—C7—C8	111.5 (2)	H28A—C28—H28C	109.5
N2—C7—C6	125.0 (2)	H28B—C28—H28C	109.5
C8—C7—C6	123.5 (2)	C26—C29—H29A	109.5
C10—C8—C7	106.7 (2)	C26—C29—H29B	109.5
C10—C8—N1	128.7 (2)	H29A—C29—H29B	109.5
C7—C8—N1	124.6 (2)	C26—C29—H29C	109.5
N1—C9—H9A	109.5	H29A—C29—H29C	109.5
N1—C9—H9B	109.5	H29B—C29—H29C	109.5
H9A—C9—H9B	109.5	C32—C31—H31A	109.5
N1—C9—H9C	109.5	C32—C31—H31B	109.5
H9A—C9—H9C	109.5	H31A—C31—H31B	109.5
H9B—C9—H9C	109.5	C32—C31—H31C	109.5
N3—C10—C8	104.7 (2)	H31A—C31—H31C	109.5
N3—C10—C11	126.3 (2)	H31B—C31—H31C	109.5
C8—C10—C11	128.9 (2)	C31—C32—C33	97.9 (8)
C10—C11—H11A	109.5	C31—C32—H32A	112.2
C10—C11—H11B	109.5	C33—C32—H32A	112.2
H11A—C11—H11B	109.5	C31—C32—H32B	112.2
C10—C11—H11C	109.5	C33—C32—H32B	112.2
H11A—C11—H11C	109.5	H32A—C32—H32B	109.8
H11B—C11—H11C	109.5	C33^i^—C33—C32	98.9 (8)
C17—C12—C13	120.2 (2)	C33^i^—C33—H33A	112.0
C17—C12—N3	120.0 (2)	C32—C33—H33A	112.0
C13—C12—N3	119.7 (2)	C33^i^—C33—H33B	112.0
C14—C13—C12	119.4 (2)	C32—C33—H33B	112.0
C14—C13—H13	120.3	H33A—C33—H33B	109.7
C12—C13—H13	120.3	C35—C34—H34A	109.5
C13—C14—C15	121.5 (2)	C35—C34—H34B	109.5
C13—C14—H14	119.2	H34A—C34—H34B	109.5
C15—C14—H14	119.2	C35—C34—H34C	109.5
C16—C15—C14	117.5 (2)	H34A—C34—H34C	109.5
C16—C15—C18	123.9 (2)	H34B—C34—H34C	109.5
C14—C15—C18	118.5 (2)	C36—C35—C34	101.0 (8)
C17—C16—C15	121.3 (2)	C36—C35—H35A	111.6
C17—C16—H16	119.4	C34—C35—H35A	111.6
C15—C16—H16	119.4	C36—C35—H35B	111.6
C16—C17—C12	120.0 (2)	C34—C35—H35B	111.6
C16—C17—H17	120.0	H35A—C35—H35B	109.4
C12—C17—H17	120.0	C35—C36—C36^i^	102.5 (8)
C19—C18—C15	128.5 (2)	C35—C36—H36A	111.3
C19—C18—H18	115.7	C36^i^—C36—H36A	111.3
C15—C18—H18	115.7	C35—C36—H36B	111.3
C18—C19—O3	122.6 (2)	C36^i^—C36—H36B	111.3
C18—C19—C20	121.9 (2)	H36A—C36—H36B	109.2
			
O1—S1—N1—C8	−66.4 (2)	N1—C8—C10—C11	−2.4 (5)
O2—S1—N1—C8	163.78 (18)	N2—N3—C12—C17	−144.2 (2)
C1—S1—N1—C8	47.0 (2)	C10—N3—C12—C17	37.6 (4)
O1—S1—N1—C9	159.4 (2)	N2—N3—C12—C13	33.9 (3)
O2—S1—N1—C9	29.6 (3)	C10—N3—C12—C13	−144.3 (3)
C1—S1—N1—C9	−87.2 (3)	C17—C12—C13—C14	0.7 (4)
C7—N2—N3—C10	−2.0 (3)	N3—C12—C13—C14	−177.4 (2)
C7—N2—N3—C12	179.5 (2)	C12—C13—C14—C15	0.1 (4)
O1—S1—C1—C2	−98.2 (2)	C13—C14—C15—C16	−1.2 (4)
O2—S1—C1—C2	32.5 (3)	C13—C14—C15—C18	177.4 (2)
N1—S1—C1—C2	147.9 (2)	C14—C15—C16—C17	1.6 (4)
O1—S1—C1—C6	76.6 (2)	C18—C15—C16—C17	−177.0 (2)
O2—S1—C1—C6	−152.7 (2)	C15—C16—C17—C12	−0.9 (4)
N1—S1—C1—C6	−37.3 (2)	C13—C12—C17—C16	−0.3 (4)
C6—C1—C2—C3	−2.5 (4)	N3—C12—C17—C16	177.8 (2)
S1—C1—C2—C3	172.1 (2)	C16—C15—C18—C19	10.3 (4)
C1—C2—C3—C4	1.7 (4)	C14—C15—C18—C19	−168.2 (3)
C2—C3—C4—C5	1.0 (4)	C15—C18—C19—O3	−0.1 (4)
C3—C4—C5—C6	−2.9 (4)	C15—C18—C19—C20	178.9 (2)
C4—C5—C6—C1	2.1 (4)	C18—C19—C20—O4	−179.2 (3)
C4—C5—C6—C7	−179.7 (2)	O3—C19—C20—O4	−0.1 (4)
C2—C1—C6—C5	0.6 (4)	C18—C19—C20—C21	0.3 (4)
S1—C1—C6—C5	−174.1 (2)	O3—C19—C20—C21	179.4 (2)
C2—C1—C6—C7	−177.7 (2)	O4—C20—C21—C26	86.3 (3)
S1—C1—C6—C7	7.6 (3)	C19—C20—C21—C26	−93.1 (3)
N3—N2—C7—C8	1.6 (3)	O4—C20—C21—C22	−94.7 (3)
N3—N2—C7—C6	−177.3 (2)	C19—C20—C21—C22	85.9 (3)
C5—C6—C7—N2	15.8 (4)	C26—C21—C22—C23	1.6 (4)
C1—C6—C7—N2	−166.0 (2)	C20—C21—C22—C23	−177.4 (2)
C5—C6—C7—C8	−162.9 (3)	C26—C21—C22—C27	−179.3 (2)
C1—C6—C7—C8	15.3 (4)	C20—C21—C22—C27	1.7 (4)
N2—C7—C8—C10	−0.7 (3)	C21—C22—C23—C24	−0.3 (4)
C6—C7—C8—C10	178.2 (2)	C27—C22—C23—C24	−179.4 (3)
N2—C7—C8—N1	179.2 (2)	C22—C23—C24—C25	−1.1 (4)
C6—C7—C8—N1	−1.9 (4)	C22—C23—C24—C28	179.6 (3)
C9—N1—C8—C10	−78.1 (4)	C23—C24—C25—C26	1.3 (4)
S1—N1—C8—C10	147.0 (2)	C28—C24—C25—C26	−179.5 (3)
C9—N1—C8—C7	102.0 (3)	C24—C25—C26—C21	−0.1 (4)
S1—N1—C8—C7	−32.9 (3)	C24—C25—C26—C29	179.8 (3)
N2—N3—C10—C8	1.6 (3)	C22—C21—C26—C25	−1.4 (4)
C12—N3—C10—C8	180.0 (2)	C20—C21—C26—C25	177.6 (2)
N2—N3—C10—C11	−176.4 (2)	C22—C21—C26—C29	178.7 (3)
C12—N3—C10—C11	1.9 (4)	C20—C21—C26—C29	−2.4 (4)
C7—C8—C10—N3	−0.6 (3)	C31—C32—C33—C33^i^	167.7 (17)
N1—C8—C10—N3	179.6 (3)	C34—C35—C36—C36^i^	−43 (3)
C7—C8—C10—C11	177.4 (3)		

Symmetry codes: (i) −*x*+2, −*y*, −*z*+1.

### Hydrogen-bond geometry (Å, °)

**Table:**

*D*—H···*A*	*D*—H	H···*A*	*D*···*A*	*D*—H···*A*
O3—H3O···O2^ii^	0.84	2.15	2.854 (3)	141.
O3—H3O···O4	0.84	2.18	2.646 (3)	115.
C3—H3···O1^iii^	0.95	2.56	3.328 (3)	138.
C23—H23···O1^iv^	0.95	2.58	3.436 (4)	150.
C9—H9C···O2	0.98	2.46	2.825 (4)	102.
C16—H16···O3	0.95	2.28	2.907 (3)	123.

Symmetry codes: (ii) −*x*+3/2, *y*−1/2, −*z*+1/2; (iii) *x*+1, *y*, *z*; (iv) −*x*+1, −*y*+1, −*z*+1.
